# A sequence-based multiple kernel model for identifying DNA-binding proteins

**DOI:** 10.1186/s12859-020-03875-x

**Published:** 2021-05-31

**Authors:** Yuqing Qian, Limin Jiang, Yijie Ding, Jijun Tang, Fei Guo

**Affiliations:** 1 School of Electronic and Information Engineering, Suzhou University of Science and Technology, Suzhou, People’s Republic of China; 2 Shenzhen Institute of Advanced Technology, Chinese Academy of Sciences, 1068 Xueyuan Avenue, Shenzhen University Town, Shenzhen, People’s Republic of China; 3 School of Computer Science and Engineering, Central South University, Changsha, People’s Republic of China

**Keywords:** DNA-binding proteins, Feature extraction, Multiple kernel learning, Support vector machine, Centered kernel alignment

## Abstract

**Background:**

DNA-Binding Proteins (DBP) plays a pivotal role in biological system. A mounting number of researchers are studying the mechanism and detection methods. To detect DBP, the tradition experimental method is time-consuming and resource-consuming. In recent years, Machine Learning methods have been used to detect DBP. However, it is difficult to adequately describe the information of proteins in predicting DNA-binding proteins. In this study, we extract six features from protein sequence and use Multiple Kernel Learning-based on Centered Kernel Alignment to integrate these features. The integrated feature is fed into Support Vector Machine to build predictive model and detect new DBP.

**Results:**

In our work, date sets of PDB1075 and PDB186 are employed to test our method. From the results, our model obtains better results (accuracy) than other existing methods on PDB1075 ($84.19\%$) and PDB186 ($83.7\%$), respectively.

**Conclusion:**

Multiple kernel learning could fuse the complementary information between different features. Compared with existing methods, our method achieves comparable and best results on benchmark data sets.

## Background

DNA-Binding Protein (DBP) plays a vital role in the function of various biomolecules, containing DNA transcription and replication. To detect DNA-binding protein via biological assays, researchers usually employed electrophoretic mobility shift assay, chromatin immunoprecipitation, Yeast One-hybrid System (Y1H) and X-ray crystallography. However, above methods are still time consuming and extremely expensive. The machine learning-based methods have been developed to solve the problem of detecting DNA-binding protein [1–3].

In the identification study of DNA-binding proteins, the main task is to determine an unknown protein whether it can bind to DNA. In the previous works, many researchers detected DBP based on structural information. Nimrod et al. [4] constructed a random forest prediction model for DNA-binding protein recognition using the average surface electrostatic potential, dipole moment, and amino acid conservation pattern information; Bhardwaj et al. [5] used overall charge, surface patches and composition feature to train a predictive model via Support Vector Machine (SVM) [6]. Ahmad et al. [7] trained a neural network model to predict DBP. The feature of protein contained the net charge of the protein, electric dipole moment and fourth moment tensor.

The number of protein sequences is larger than the number of known protein structures. The number of protein with relevant structural information is very low and most of the proteins do not have the corresponding structural information. Therefore, the structure-based models cannot be widely used to detect DBP. A method based on protein sequence [8] constructed a Support Vector Machine (SVM) model with amino acid composition and materialized property information. Liu and Cai et al. [9–11] extracted overall amino acid composition and Pseudo Amino Acid Composition (PseAAC) to represent protein feature. Liu et al. [12] developed a model called iDNAPro-PseAAC, which is extended with evolutionary information of protein sequence. Kumar et al. [13] used Position Specific Scoring Matrix (PSSM) to propose a classifier called DNAbinder, which is based on SVM. PSSM was produced via PSI-BLAST software [14], which could obtain evolutionary conservation information. The Local-DPP [1] captured local conservation information of PSSM and trained an ensemble model to predict DBP. DBPPred [15] employed Random Forest (RF) to get the optimal feature subset and trained Gaussian Naive Bayes model for predicting DBP. Zou et al. utilized a Fuzzy Kernel Ridge Regression model with Multi-View Sequence Features (FKRR-MVSF) [16] to predict DBP. To further improve the accuracy of DBP prediction, Ding et al. [17] employed a Multi-Kernel SVM based on Heuristically Kernel Alignment (MKSVM-HKA) to integrate different features from protein sequence. In addition, a multiple kernel-based fuzzy SVM model [18] of DNA-binding proteins also was developed to improve prediction performance. Liu et al. [19] proposed a stacking framework model for predicting DBP by orchestrating multi-view features. This stacking framework model was named as MSFBinder. Rahman et al. [20] developed a DNA-binding Protein Prediction model using Chou general PseAAC (DPP-PseAAC) and SVM based Recursive Feature Elimination (RFE) approach. Adilina et al. [21] extracted several features via PseAAC and carried out two different types of feature selection to build predictive model of DBP.

In practical applications, the sequence-based approaches are more adaptable. DNA-methylation sites, recombination spots, Post Translational Modification (PTM) sites (protein) and Protein-Protein Interactions (PPI) have been predicted by sequential methods. In recent years, machine learning methods have been widely used in bioinformatics [16, 17, 22–38]. And some of the biological problems are solved very well, including O-GlcNAcylation sites [23], protein subcellular localization [25, 39, 40], Methyladenosine Sites [22, 26], drug-target interactions [27–31, 37, 41], drug-drug interactions [42, 43], lncRNA-Protein interaction [35, 36] protein crystallization prediction [32, 44], potential disease-associated microRNAs [24, 33, 34, 45, 46] and other RNAs [47–50].

Inspired by the previous work [1, 8, 9, 11, 13, 16, 17], we propose a new predictive model for DNA-binding protein through multi-kernel support vector machine. Firstly, several types of features are extracted from protein sequences. And these features are employed to construct kernel matrices. We use Multi-Kernel Learning-based on Centered Kernel Alignment (MKL-CKA) algorithm to combine these kernels and obtain an integrated kernel for training SVM model. We call this model as Multi-Kernel SVM (MKSVM) model. Finally, MKSVM is utilized to detect new DNA-binding proteins. Compared with other state-of-the-art models, the proposed method achieves better results. The accuracy of our model are $84.19\%$ and $83.7\%$ on the PDB1075 (leave one out test) and PDB186 (independent test) data sets, respectively.

## Results

In this section, we test our method on PDB1075 and PDB186 data sets. Firstly, we perform a Leave One Out Cross validation (LOOCV) on the PDB1075. Next, our model are trained by the PDB1075 and tested on the PDB186. Other existing methods are also test on PDB1075 and PDB186. The data set and source code (with Python Programming Language) is obtained from https://figshare.com/s/cf56cef6659c7eed16c9.

### Data sets

The details of PDB1075 and PDB186 data sets are list in Table 1. The benchmark data sets (PDB1075 and PDB186) are selected from Protein Data Bank (PDB) [51]. Any two sequences have not more than $25\%$ similarity. Protein sequences which less than 50 amino acids or contain the ‘X’ character must be removed. The PDB1075 data set (constructed by Liu et al. [9]) is used to test our model under LOOCV. The PDB186 data set (constructed by Lou et al. [15]) is used for independent testing.

**Table 1:** The detail information of two benchmark data sets

Data sets	PDB1075	PDB186
Number of positive	525	93
Number of negative	550	93
Number of total sample	1075	186

**Table 2:** The ACC of different parameter values on PDB1075 (five-fold cross validation)

Parameter values	ACC (%)	
	*lag*_max_ for PsePSSM	*lg* for NMBAC
5	74.66	66.81
10	77.78	68.24
15	77.02	69.95
20	76.88	70.79
25	77.63	71.03
30	77.21	71.09
35	76.94	71.00
40	77.56	70.91
45	77.71	70.86

### Measurements

The main measures for the evaluation of performance are Accuracy (ACC), Matthew’s Correlation Coefficient (MCC), Sensitivity (SN), Specificity (SP), and Area Under ROC (AUC). The calculation formulas of ACC, SN, SP and MCC indicators are calculated as follows:

1a $$\begin{aligned} ACC&=\frac{TP+TN}{TP+FP+TN+FN} \end{aligned}$$

1b $$\begin{aligned} SN&=\frac{TP}{TP+FN} \end{aligned}$$

1c $$\begin{aligned} Spec&=\frac{TN}{TN+FP} \end{aligned}$$

1d $$\begin{aligned} MCC&=\frac{TP \times TN - FP \times FN}{\sqrt{(TP+FN)\times (TN+FP) \times (TP+FP) \times (TN+FN)}} \end{aligned}$$

where *TP* is the correct number of positive samples, *TN* is the correct number of negative samples, *FN* is the number of false negative samples and *FP* is the number of false positive samples. Area Under of receiver operating characteristic Curve (AUC) is obtained by calculating the area under the Receiver Operating characteristic Curve (ROC). The higher value of AUC, the better predictive effect.

### Parameters selection

To achieve the best performance, we need to select optimal parameters of predictive model. In this section, we employ grid search method to select optimal parameters for SVM model.

#### The parameters selection of features

To select the optimal parameters of feature NMBAC and PsePSSM, we test the different parameters (the max value of $lag_{max}$ and *lg* for PsePSSM and NMBAC) under five-fold cross validation (on PDB1075 data set). We set the range of *lg* (NMBAC) and $lag_{max}$ (PsePSSM) values from 5 to 45 (step of 5). In Table 2, the results of the prediction show that the optimal *lg* (NMBAC) as 30 and $lag_{max}$ (PsePSSM) as 10 in this study.

#### Selection of *C* and $\boldsymbol{\gamma}$  

For the selection of SVM parameters, we use the grid search method and the 5-fold Cross Validation (5-CV) method. We set the range of parameter from $2^{-5}$ to $2^{5}$ with step $2^{1}$. The optimal parameters of results are show in Table 3.

**Table 3:** The optimal parameters for SVM (single kernel)

Feature	***C***	***γ***
GE	$2^{0}$	$2^{0}$
MCD	$2^{3}$	$2^{-5}$
NMBAC	$2^{-1}$	$2^{-1}$
PSSM-AB	$2^{0}$	$2^{-4}$
PSSM-DWT	$2^{1}$	$2^{-5}$
PsePSSM	$2^{1}$	$2^{-5}$

**Table 4:** The performance of different kernels (RBF kernel) on PDB1075 data set (leave one out)

Kernel type	Model	ACC (%)	SN (%)	Spec (%)	MCC	AUC
${\mathbf {K}}_{GE}$	SVM	71.6	70.1	73.1	0.432	0.785
${\mathbf {K}}_{MCD}$	SVM	70.9	68.2	73.5	0.417	0.761
${\mathbf {K}}_{NMBAC}$	SVM	71.1	73.3	69.1	0.424	0.771
${\mathbf {K}}_{PSSM-AB}$	SVM	76.9	84.4	69.8	0.547	0.839
${\mathbf {K}}_{PSSM-DWT}$	SVM	76.0	79.2	72.9	0.522	0.837
${\mathbf {K}}_{PsePSSM}$	SVM	78.5	82.5	74.7	0.573	0.857
Mean weighted kernels	SVM	83.1	84.6	81.8	0.664	0.913
MKL-CKA	SVM	**84.2**	**85.9**	**82.6**	**0.684**	**0.914**

The bold font indicates the largest value in the column

**Table 5:** The weight of six kernels (RBF kernel) by MKL-CKA

Kernel type	Kernel weights
${\mathbf {K}}_{GE}$	0.165
${\mathbf {K}}_{MCD}$	0.112
${\mathbf {K}}_{NMBAC}$	0.135
${\mathbf {K}}_{PSSM-AB}$	0.219
${\mathbf {K}}_{PsePSSM}$	0.114
${\mathbf {K}}_{PSSM-DWT}$	0.254

**Table 6:** The sensitivity of different kernels (features) on PDB1075 data set (under the specificity of 0.5)

Kernel type	Sensitivity
${\mathbf {K}}_{GE}$	0.8857
${\mathbf {K}}_{MCD}$	0.8495
${\mathbf {K}}_{NMBAC}$	0.8590
${\mathbf {K}}_{PSSM-AB}$	0.9352
${\mathbf {K}}_{PsePSSM}$	0.9523
${\mathbf {K}}_{PSSM-DWT}$	0.9657
Mean weighted kernels	0.9847
MKL-CKA	0.9885

**Fig. 1:**
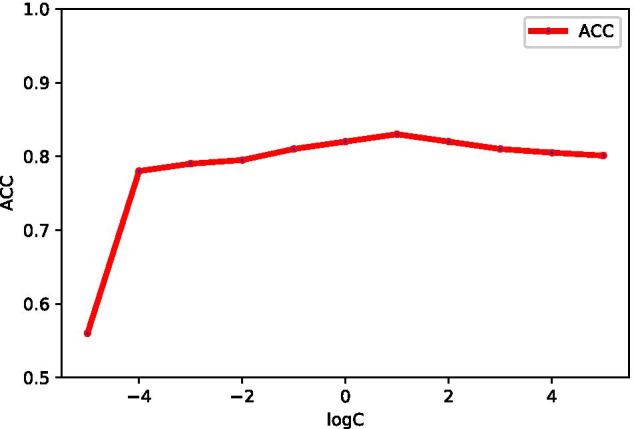
The ACC values under parameters of *C* on PDB1075 data set (five-fold cross validation)

Before combining multiple kernels, the parameter $\gamma$ for 6 types of kernels are obtained from their single kernels (Table 3). To achieve the optimal parameters of *C* under MKSVM (average weight for each kernel), we also utilize the above *C* range. Comparing the accuracy of different *C* values, the corresponding values of ACC are shown in the Fig. 1. When $C=2 \ (logC=1)$, the MKSVM (average weight for each kernel) achieves best ACC ($82.8\%$). In our study, the parameter (*C*) of MKSVM (with MKL-CKA) is same as MKSVM with mean weighted.

**Fig. 2:**
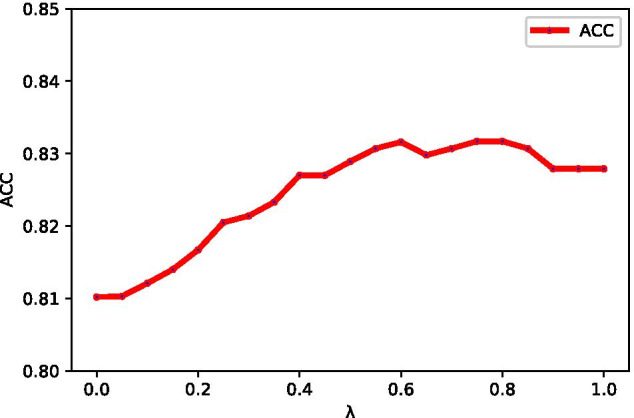
The ACC values under parameters of $\lambda$ on PDB1075 data set (five-fold cross validation)

To obtain the optimal parameter ($\lambda$) of MKL-CKA, we try the different value of $\lambda$ from 0 to 1 (step is 0.05) under 5-CV on PDB1075 data set. The results are shown in the Fig. 2. When $\lambda = 0.8$, the ACC value is the highest. We set 0.8 as the optimal parameter ($\lambda$) of MKL-CAK.

### Performance analysis on PDB1075

**Fig. 3:**
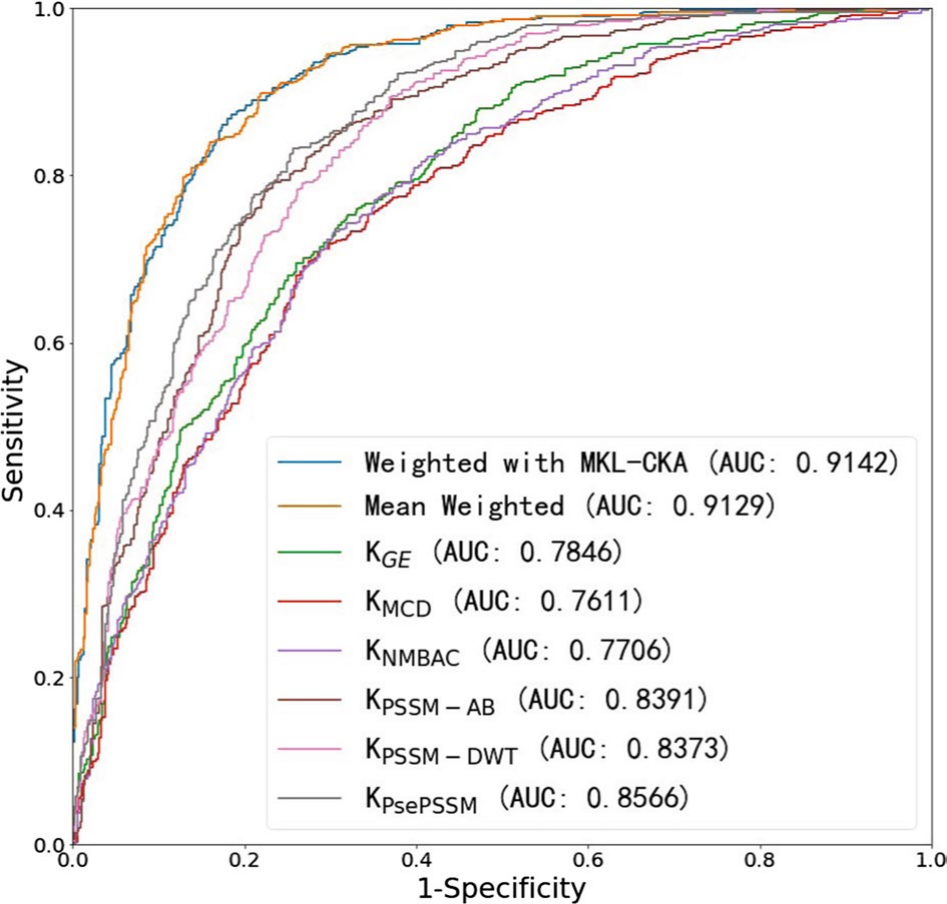
The ROC comparison of different kernels (feature) via Leave one out test on PDB1075 data set

We test the performance of different kernels (features) on PDB1075 (under LOOCV). The results are shown in Table 4 and Fig. 3.

As we can see from the table, the results of multi-kernel learning are much better than single kernel model. The PSSM-AB (MCC: 0.547), PSSM-DWT (MCC: 0.522) and PsePSSM (MCC: 0.573) kernels with PSSM information are better than those of GE (MCC: 0.432), MCD (MCC: 0.417) and NMBAC (MCC: 0.424). Among them, we calculate the weights of six kernels by MKL-CKA method (Table 5). The integrated kernel (with MKL-CKA) has the highest results in ACC ($84.2\%$), MCC (0.684), SN ($85.9\%$), SP ($82.6\%$) and AUC (0.914). Obviously, the integrated kernel (with MKL-CKA) is higher than mean weighted kernel.

Under the specificity of 0.5 (on PDB1075 data set), the sensitivity values of different kernel are following: ${\mathbf {K}}_{GE}$: 0.8857, ${\mathbf {K}}_{MCD}$: 0.8495, ${\mathbf {K}}_{NMBAC}$: 0.8590, ${\mathbf {K}}_{PSSM-AB}$: 0.9352, ${\mathbf {K}}_{PsePSSM}$: 0.9657, ${\mathbf {K}}_{PSSM-DWT}$: 0.9523, mean weighted kernel: 0.9847, and ${\mathbf {K}}_{MKL-CKA}$: 0.9885. Some kernels have bias in the learning process. MKL-CKA could filter noise kernels (reducing bias of kernels) by setting low weights of kernels. And the sensitivity of MKL-CKA (0.9885) is better than best single kernel (${\mathbf {K}}_{PSSM-AB}$: 0.9352). Although our MKL algorithm only improves sensitivity value with a few percentage points, the purpose of MKL is to filter noise feature (kernel) and integrate multiple effective features. The Table 6 shows the sensitivity of different kernels (features) on PDB1075 data set (Under the specificity of 0.5).

**Table 7:** The running time of different kernels (features) on PDB1075 data set (training)

Kernel type	Sec
${\mathbf {K}}_{GE}$	0.418
${\mathbf {K}}_{MCD}$	3.79
${\mathbf {K}}_{NMBAC}$	0.627
${\mathbf {K}}_{PSSM-AB}$	0.678
${\mathbf {K}}_{PsePSSM}$	3.7
${\mathbf {K}}_{PSSM-DWT}$	3.47
Mean weighted kernels	28.7
MKL-CKA	68

**Table 8:** The performance of different kernel functions on PDB1075 data set (Five-fold cross validation)

Feature	ACC (%)			
	Linear kernel	Polynomial kernel	RBF kernel	Sigmoid kernel
GE	69.30	68.18	69.97	69.76
MCD	69.39	70.04	70.21	62.14
NMBAC	72.04	72.91	71.01	70.97
PSSM-AB	75.34	75.72	76.54	60.01
PSSM-DWT	73.86	71.25	76.26	66.12
PsePSSM	77.32	77.64	78.36	76.01
MKL-CKA	81.39	78.79	83.01	72.34

We also evaluate the running time of different models with different kernels. The results are shown in Table 7. The programs are carried out on the computer Intel Core i5 3.2 GHz CPU 8 GB RAM. The running time (s) of our methods are ${\mathbf {K}}_{GE}$: 0.418, ${\mathbf {K}}_{MCD}$: 3.79, ${\mathbf {K}}_{NMBAC}$: 0.627, ${\mathbf {K}}_{PSSM-AB}$: 0.678, ${\mathbf {K}}_{PsePSSM}$: 3.7, ${\mathbf {K}}_{PSSM-DWT}$: 3.47, mean weighted kernel: 28.7, and MKL-CKA: 68, respectively. Because multiple kernel matrices are calculated and the weight value of each kernel matrix is estimated, MKL-CKA is the most time-consuming.

What’s more, other kernel functions (e.g. linear kernel, polynomial kernel, and sigmoid kernel) are also test. We compare RBF kernel with other 3 types of kernel functions under five-fold cross validation. The results are list in Table 8, which shows that RBF kernel obtain better ACC on GE ($69.97\%$), MCD ($70.21\%$), PSSM-AB ($76.54\%$), PSSM-DWT ($76.26\%$) and PsePSSM ($78.36\%$), respectively. MKL-CKA also is employed to combine 6 features with four kernel functions, respectively. The RBF kernel (with MKL-CKA) achieves best ACC ($83.01\%$).

### Comparison to existing predictors on PDB1075

**Table 9:** Compared with existing methods on PDB1075 data set (LOOCV)

Methods	ACC (%)	MCC	SN (%)	Spec (%)
IDNA-Prot|dis	77.30	0.54	79.40	75.27
PseDNA-Pro	76.55	0.53	79.61	73.63
IDNA-Prot	75.40	0.50	83.81	64.73
DNA-Prot	72.55	0.44	82.67	59.76
DNAbinder	73.95	0.48	68.57	79.09
iDNAPro-PseAAC	76.56	0.53	75.62	77.45
Kmer1+ACC	75.23	0.50	76.76	73.76
Local-DPP	79.10	0.59	84.80	73.60
MKSVM-HKA	81.30	0.63	82.29	80.36
MSFBinder	83.35	0.67	83.62	**83.09**
FKRR-MVSF	83.26	0.67	85.71	80.91
Our method (MKSVM with MKL-CKA)	**84.19**	**0.68**	**85.91**	82.55

The bold font indicates the largest value in the column

The MKSVM (with MKL-CKA) model and other methods are also test on PDB1075 data set (under LOOCV). The results of ACC, MCC, SN and SP are list in Table 9. Existing methods include IDNA-Prot|dis [2], DNAbinder [13], iDNAPro-PseAAC [10], Kmer1+ACC [12], iDNA-Prot [52], DNA-Prot [53], PseDNA-Pro [9], MKSVM-HKA [17], MSFBinder [19], FKRR-MVSF [16] and Local-DPP [1]. Among these methods, MKSVM-HKA (MCC: 0.63), MSFBinder (MCC: 0.67), FKRR-MVSF (MCC: 0.67), iDNA Pro-PseAAC (MCC: 0.53), PseDNA-Pro (MCC: 0.53), IDNA-Prot|dis (MCC: 0.54) and Local-DPP (MCC: 0.59) also obtained good performance. Local-DPP and iDNAPro-PseAAC take advantage of the PSSM feature to improve performance. MKSVM-HKA, FKRR-MVSF and MSFBinder employed MKL algorithm and ensemble strategy to integrate multiple information and further improve the predictive accuracy. Our method (MKSVM with MKL-CKA) is also based on MKL and achieves best MCC (0.68). Although, the SP value of MSFBinder ($83.09\%$) is higher than our method ($82.55\%$). Our method is the highest in ACC ($84.19\%$), MCC (0.68), SN ($85.91\%$).

The statistical significance tests of the differences is necessary. The results in Table 10 list that our method make statistically significant improvement over the other methods (*P*-value $<0.05$, by *t*-test, in term of MCC). The comparison is under 10 fold cross validation on PDB1075. The difference between Local-DPP and our method is significant (*P*-value: 6.0421E$-$6). Comparing with MKSVM-HKA (*P*-value: 1.5438E$-$4), MSFBinder (*P*-value: 0.0098) and FKRR-MVSF (*P*-value: 0.0103), our method also shows significantly better prediction accuracy.

**Table 10:** The statistics of different methods

Methods	***P*** value
Local-DPP	6.0421E-6
MKSVM-HKA	1.5438E-4
MSFBinder	0.0098
FKRR-MVSF	0.0103

**Table 11:** The results of comparison between MKSVM (with MKL-CKA) model and other existing methods on PDB186 data set (independent test)

Methods	ACC (%)	MCC	SN (%)	Spec (%)
IDNA-Prot|dis	72.0	0.445	79.5	64.5
IDNA-Prot	67.2	0.344	67.7	66.7
DNA-Prot	61.8	0.240	69.9	53.8
DNAbinder	60.8	0.216	57.0	64.5
DBPPred	76.9	0.538	79.6	**74.2**
iDNAPro-PseAAC	71.5	0.442	82.8	60.2
Kmer1+ACC	71.0	0.431	82.8	59.1
Local-DPP	79.0	0.625	92.5	65.6
DPP-PseAAC	77.4	0.550	83.0	70.9
Adilina’s work	82.3	0.670	95.0	69.9
MKSVM-HKA	81.2	0.648	94.6	67.7
MSFBinder	79.6	0.616	93.6	65.6
FKRR-MVSF	81.7	0.676	**98.9**	64.5
RF*	79.0	0.593	89.3	68.8
FNN*	75.3	0.520	87.1	63.4
Our method* (MKSVM with MKL-CKA)	**83.7**	**0.691**	93.6	**74.2**

The bold font indicates the largest value in the column

* The model is built via the 6 types of our features

### Independent test

In order to further evaluate the performance of MKSVM (with MKL-CKA) model, we use PDB1075 to construct MKSVM model and test it via PDB186 data set. The results of comparison are shown in Table 11.

Our method achieves $83.7\%$, 0.691, $93.6\%$, and $74.2\%$ on ACC, MCC, SN, and SP, respectively. From the results of independent test, we can find out that our method has certain accuracy in the prediction of DBP. Adilina’s work (MCC: 0.670), MKSVM-HKA (MCC: 0.648), MSFBinder (MCC: 0.616) and FKRR-MVSF (MCC: 0.676) obtained good results on PDB186. Adilina et al. [21] employed 7 types of features and the strategy of feature selection to construct predictive model. FKRR-MVSF [16] and MKSVM-HKA [17] utilized MKL algorithm to combine several features. MSFBinder [19] built a stacking framework model by multiple features. The multiple information fusion-based methods achieved better results. Our method (MKSVM with MKL-CKA) performs better (MCC: 0.691) than most of existing models on PDB186 data set. From the results, the fusion of multiple information can improve the performance of the prediction model. FKRR-MVSF (MCC: 0.676), MKSVM-HKA (MCC: 0.648) and MSFBinder (MCC: 0.616) achieved better results on PDB186. We also test the performance of Random Forest (RF) and Feed forward Neural Network (FNN) on PDB186. RF and FNN achieve MCC of 0.593 and 0.520, respectively. SVM can achieve better performance on small data sets.

## Discussion

How to describe and integrate the information of proteins is the difficulty in predicting DNA-binding proteins. In our study, MKL-CKA is utilized to integrate 6 types of features and achieves better results on PDB1075 (MCC: 0.68) and PDB186 (MCC: 0.69) data sets. Other methods, such as FKRR-MVSF, MKSVM-HKA, MSFBinder and Adilina’s work, also obtained good performance. We can find that multiple information fusion-based methods have better generalization performance on DBP prediction. To obtain the optimal weights of kernels, MKL-CKA maximizes the alignment score between feature space and label space. Ideal kernel (label space) contains the category information of the training samples. The Laplace smooth term can further optimize weight values. The performance of MKL-CKA (MCC: 0.684) is better than mean weighted kernels (MCC: 0.664) on PDB1075 (LOOCV). The process of MKL is similar to feature selection. MKL weights each kernel matrix (6 types of features). Whether the predictive models are based on MKL or feature selection, the noise features can be effectively filtered.

## Conclusion

Although many models have been constructed to predict DBP, they can still be optimized to improve accuracy. Existing methods do not consider the removal of outliers in data sets. In the future, we will filter noise samples and improve the predictive accuracy of DBP by fuzzy theory and ensemble strategy.

## Methods

**Fig. 4:**
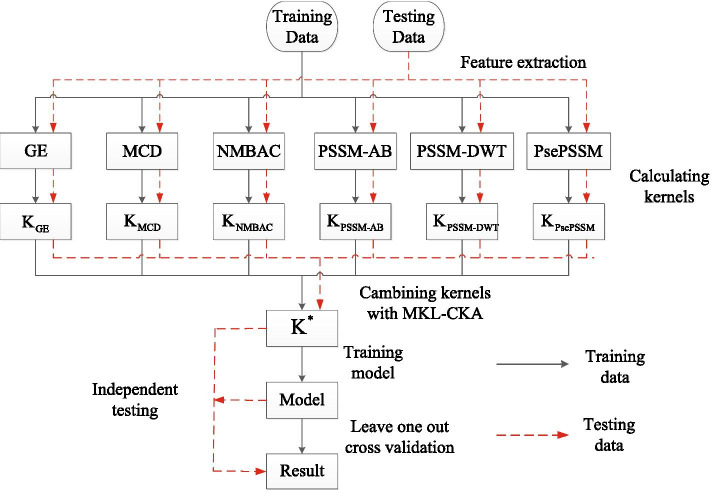
The framework of our method

DBP identification can be considered as a traditional binary classification problem, and we use SVM algorithm to construct predictive model. First, we extract the features of the protein from the sequence information. Six types of kernel matrices are constructed from these features. Above kernels are integrated to construct optimal kernel (including training kernel and testing kernel) by Multi-Kernel Learning-based on Centered Kernel Alignment (MKL-CKA) algorithm. We employ the combined kernel to build a SVM model and identify DBP. Figure 4 represents the framework of MKLSVM (with MKL-CKA). Firstly, six types of features are extracted from protein sequences. Then, six kernels are built by Radial Basis Function (RBF). MKL-CKA algorithm combines the 6 types of kernels. Next, we use the combined kernel and SVM algorithm construct the final predictive model to detect DBP.

### Sequence feature

There are six types of features from protein sequence information, including PSSM-based Discrete Wavelet Transform (PSSM-DWT) [54], PSSM-based Average Blocks (PSSM-AB) [55], Pseudo-PSSM (PsePSSM) [10, 12, 56, 57], Multi-scale Continuous and Discontinuous descriptor (MCD) [58], Global Encoding (GE) [59] and Normalized Moreau-Broto Auto correlation (NMBAC) [60, 61]. These features have been detailed descripted in related literatures. We employ RBF to construct six types of kernels. The function formula of RBF is as follow:

2 $$\begin{aligned} K_{ij}=K({\mathbf {x}}_{i},{\mathbf {x}}_{j}) = exp(-\gamma \Vert {\mathbf {x}}_{i} - {\mathbf {x}}_{j} \Vert ^{2}), \ i,j=1,2,...,N \end{aligned}$$

where $\gamma$ is the kernel bandwidth. We can obtain a kernel set ${\mathbf {K}}$ as follows:

3 $$\begin{aligned} {\mathbf {K}}= \left\{ {\mathbf {K}}_{GE}, {\mathbf {K}}_{MCD}, {\mathbf {K}}_{NMBAC}, {\mathbf {K}}_{PSSM-AB}, {\mathbf {K}}_{PSSM-DWT}, {\mathbf {K}}_{PsePSSM} \right\} \end{aligned}$$

### Support vector machine

Support Vector Machine (SVM) is a classification algorithm, which is developed by Vapnik [6]. By finding the optimal hyper plane, the data set is separated on positive and negative points. The instance-label pairs (a training sample) {${\mathbf {x}}_{i},y_{i}$}, ${\mathbf {x}}_{i}\in {\mathbf {R}}^{d \times 1}$ and $i=1,2,...,N$. Labels $y_{i}\in \{ +1,-1\}$. The decision function is defined as following:

4 $$\begin{aligned} f({\mathbf {x}}) = sign[\sum _{i=1}^N y_{i}\alpha _{i}\cdot K({\mathbf {x}},{\mathbf {x}}_{i})+b] \end{aligned}$$

The coefficient $\pmb {\alpha }$ are estimated by solving a Quadratic Programming (QP) problem:

5a $$\begin{aligned}&Maximize \quad \sum _{i=1}^N \alpha _{i} - \frac{1}{2}\sum _{i=1}^N \sum _{j=1}^N \alpha _{i}\alpha _{j}\cdot y_{i}y_{j}\cdot K({\mathbf {x}}_{i},{\mathbf {x}}_{j}) \end{aligned}$$

5b $$\begin{aligned}&s.t. \quad 0 \le \alpha _{i} \le C \end{aligned}$$

5c $$\begin{aligned}&\sum _{i=1}^N \alpha _{i}y_{i} = 0, i=1,2,...,N \end{aligned}$$

${\mathbf {x}}_{i}$ is support vector when the corresponding $\alpha _{i} > 0$. *C* denotes the tradeoff between margin and misclassification error. What’s more, we construct a SVM model by LIBSVM [62](http://www.csie.ntu.edu.tw/~cjlin/libsvm/). We employ the grid search method to obtain the optimal parameters of the SVM.

### Multiple kernel learning

Because of strong theoretical guarantee and excellent experimental performance, the MKL-CKA [63, 64] method is adopted in our study. MKL-CKA is a multi-kernel learning algorithm based on kernel alignment. The optimal kernel is calculated as follows:

6a $$\begin{aligned}&{\mathbf {K}}^{*} = \sum _{i=1}^{m} \beta _{i} {\mathbf {K}}_{i}, \end{aligned}$$

6b $$\begin{aligned}&{\mathbf {K}}_{i} \in {\mathbf {R}}^{N \times N}, \end{aligned}$$

6c $$\begin{aligned}&\sum _{i=1}^{m} \beta _{i} = 1 \end{aligned}$$

where *m* is the number of kernels and $\beta _{i}$ is the weight of the kernel ${\mathbf {K}}_{i}$.

The value of kernel alignment is defined as follow:

7 $$\begin{aligned} A({\mathbf {P}},{\mathbf {Q}}) = \frac{\left\langle {\mathbf {P}},{\mathbf {Q}} \right\rangle _{F}}{\Vert {\mathbf {P}} \Vert _{F} \Vert {\mathbf {Q}} \Vert _{F}} \end{aligned}$$

where ${\mathbf {P}}, {\mathbf {Q}} \in {\mathbf {R}}^{N \times N}$, $\left\langle {\mathbf {P}},{\mathbf {Q}} \right\rangle _{F} = Trace({\mathbf {P}}^{T}{\mathbf {Q}})$ is the Frobenius inner product and $\Vert {\mathbf {P}} \Vert _{F} = \sqrt{ \left\langle {\mathbf {P}},{\mathbf {P}} \right\rangle _{F}}$ is Frobenius norm.

The score of kernel alignment can be described as the cosine similarity between two kernels. The more high score of kernel alignment, the greater similarity between the kernels. We hope that the alignment score between combined kernel (feature space) and ideal kernel (label space) is high. So, the function formula of centered kernel alignment is as follow:

8a $$\begin{aligned}&\underset{\pmb {\beta } \ge 0}{\text{ max }} \quad CA({\mathbf {K}}^{*},{\mathbf {y}}_{train}{\mathbf {y}}_{train}^{T}) = \underset{\pmb {\beta } \ge 0}{\text{ max }}\quad \frac{\left\langle {\mathbf {U}}_{N}{\mathbf {K}}^{*}{\mathbf {U}}_{N},{\mathbf {y}}_{train}{\mathbf {y}}_{train}^{T} \right\rangle _{F}}{\Vert {\mathbf {U}}_{N}{\mathbf {K}}^{*}{\mathbf {U}}_{N} \Vert _{F} \Vert {\mathbf {y}}_{train}{\mathbf {y}}_{train}^{T} \Vert _{F}} \end{aligned}$$

8b $$\begin{aligned}&s.t. \ {\mathbf {K}}^{*} = \sum _{i=1}^{m} \beta _{i}{\mathbf {K}}_{i}, \end{aligned}$$

8c $$\begin{aligned}&\beta _{i} \ge 0, \ i = 1,2,...,m, \end{aligned}$$

8d $$\begin{aligned}&\sum _{i=1}^{m} \beta _{i} = 1 \end{aligned}$$

where the centering matrix is ${\mathbf {U}}_{N} = {\mathbf {I}}_{N} - (1/N){\mathbf {l}}_{N}{\mathbf {l}}_{N}^{T}$, ${\mathbf {U}}_{N} \in {\mathbf {R}}^{N \times N}$ is centering matrix. ${\mathbf {I}}_{N} \in {\mathbf {R}}^{n \times n}$ denotes identity matrix. ${\mathbf {l}}_{N}$ is identity vector. So, formula 8 can be written as follow:

9a $$\begin{aligned}&\underset{\mathbf {\pmb {\beta }} \ge 0}{\text{ max }} \quad \frac{\pmb {\beta }^{T}{\mathbf {a}}}{\sqrt{\pmb {\beta }^{T}{\mathbf {M}}\pmb {\beta }}} \end{aligned}$$

9b $$\begin{aligned}&s.t. \ {\mathbf {K}}^{*} = \sum _{i=1}^{m} \beta _{i}{\mathbf {K}}_{i}, \end{aligned}$$

9c $$\begin{aligned}&\beta _{i} \ge 0, \ i = 1,2,...,m, \end{aligned}$$

9d $$\begin{aligned}&\sum _{i=1}^{m} \beta _{i} = 1 \end{aligned}$$

In Eq. (9), ${\mathbf {a}}\in {\mathbf {R}}^{m \times 1}$ and ${\mathbf {M}}\in {\mathbf {R}}^{m \times m}$ is represented as Eqs. (10) and (11).

10 $$\begin{aligned} \begin{aligned} {\mathbf {a}}&= \left( \left\langle {\mathbf {U}}_{N}{\mathbf {K}}_{1}{\mathbf {U}}_{N},{\mathbf {y}}_{train}{\mathbf {y}}_{train}^{T} \right\rangle _{F} ,...,\left\langle {\mathbf {U}}_{N}{\mathbf {K}}_{m}{\mathbf {U}}_{N},{\mathbf {y}}_{train}{\mathbf {y}}_{train}^{T} \right\rangle _{F} \right) ^{T} \in {\mathbf {R}}^{m \times 1} \end{aligned} \end{aligned}$$

11a $$\begin{aligned} {\mathbf {M}}&= \left[ \begin{array}{cccc} M_{1,1} &{} M_{1,2} &{} \cdots &{} M_{1,m} \\ M_{2,1} &{} P_{2,2} &{} \cdots &{} M_{2,m} \\ \vdots &{} \vdots &{} M_{e,f} &{} \vdots \\ M_{m,1} &{} M_{m,2} &{} \cdots &{} M_{m,m} \end{array} \right] _{m \times m} \end{aligned}$$

11b $$\begin{aligned} M_{e,f}&= \left\langle {\mathbf {U}}_{N}{\mathbf {K}}_{e}{\mathbf {U}}_{N},{\mathbf {U}}_{N}{\mathbf {K}}_{f}{\mathbf {U}}_{N} \right\rangle _{F} \end{aligned}$$

11c $$\begin{aligned} e,f&=1,2,...,m \end{aligned}$$

Equation 9 also can be represented as:

12a $$\begin{aligned}&\underset{\mathbf {\beta } \ge 0}{\text{ min }} \quad \pmb {\beta }^{T}{\mathbf {M}}\pmb {\beta } - 2\pmb {\beta }^{T}{\mathbf {a}} \end{aligned}$$

12b $$\begin{aligned}&s.t. \ {\mathbf {K}}^{*} = \sum _{i=1}^{m} \beta _{i}{\mathbf {K}}_{i}, \end{aligned}$$

12c $$\begin{aligned}&\beta _{i} \ge 0, \ i = 1,2,...,m, \end{aligned}$$

12d $$\begin{aligned}&\sum _{i=1}^{m} \beta _{i} = 1 \end{aligned}$$

In order to prevent extreme situations (the weight of a kernel is close to 1 and the remaining weights are close to 0), we employ the Laplacian regular term to smooth the weights:

13 $$\begin{aligned} \begin{aligned} \sum _{i,j}^{P} (\beta _{i} - \beta _{j})^2 W_{ij}&= \sum _{i,j}^{P} (\beta _{i}^2 + \beta _{j}^2 - 2 \beta _{i} \beta _{j}) W_{ij}\\&= \sum _{i}^{P} \beta _{i}^2 D_{ii} + \sum _{j}^{P} \beta _{j}^2 D_{jj} - 2 \sum _{i,j}^{P} \beta _{i} \beta _{j} W_{ij}\\&= 2 \pmb {\beta }^{T} {\mathbf {L}} \pmb {\beta } \end{aligned} \end{aligned}$$

In Eq. (13), $i,j=1,...,m$, ${\mathbf {W}}\in {\mathbf {R}}^{m \times m}$ is the cosine similarity between two kernels. ${\mathbf {W}}$ can be calculated by Eq. (7). ${\mathbf {D}} \in {\mathbf {R}}^{m \times m}$ is a diagonal matrix, which is calculated by $D_{ii} = \sum _{j=1}^{m} W_{ij}$. ${\mathbf {L}}\in {\mathbf {R}}^{m \times m}$ is graph Laplacian matrix, which is obtained by ${\mathbf {L}} = {\mathbf {D}} -{\mathbf {W}}$. Equation (12) and formula 13 are integrated as follow:

14a $$\begin{aligned}&\underset{\mathbf {\beta } \ge 0}{\text{ min }} \quad \pmb {\beta }^{T}{\mathbf {M}}\pmb {\beta } - 2\pmb {\beta }^{T}{\mathbf {a}} + \lambda \pmb {\beta }^{T} {\mathbf {L}} \pmb {\beta }=\underset{\mathbf {\beta } \ge 0}{\text{ min }} \quad \pmb {\beta }^{T}({\mathbf {M}}+ \lambda {\mathbf {L}})\pmb {\beta } - 2\pmb {\beta }^{T}{\mathbf {a}} \end{aligned}$$

14b $$\begin{aligned}&s.t. \ {\mathbf {K}}^{*} = \sum _{i=1}^{m} \beta _{i}{\mathbf {K}}_{i}, \end{aligned}$$

14c $$\begin{aligned}&\beta _{i} \ge 0, \ i = 1,2,...,m, \end{aligned}$$

14d $$\begin{aligned}&\sum _{i=1}^{m} \beta _{i} = 1 \end{aligned}$$

where $\lambda$ is a hyper parameter of MKL-CKA. Finally, the weights obtained according to formula 14 and we calculate the optimal kernel by formula 6a.

## Data Availability

The datasets generated and/or analysed during this study are available under open licenses in the data repository, https://figshare.com/s/cf56cef6659c7eed16c9.

## Abbreviations

DBP: DNA-Binding Proteins
ML: Machine Learning
MKL-CKA: Multiple Kernel Learning-based on Centered Kernel Alignment
SVM: Support Vector Machine
Y1H: Yeast One-hybrid System
PseAAC: Pseudo Amino Acid Composition
PSSM: Position Specific Scoring Matrix
RF: Random Forest
FKRR-MVSF: Fuzzy Kernel Ridge Regression model with Multi-View Sequence Features
MKSVM-HKA: Multi-Kernel SVM based on Heuristically Kernel Alignment
DPP-PseAAC: DNA-binding Protein Prediction model using Chou general PseAAC
RFE: Recursive Feature Elimination
PTM: Post Translational Modification
PPI: Protein–Protein Interactions
MKSVM: Multi-Kernel SVM
RBF: Radial Basis Function
PSSM-DWT: PSSM-based Discrete Wavelet Transform
PSSM-AB: PSSM-based Average Blocks
PsePSSM: Pseudo-PSSM
MCD: Multi-scale Continuous and Discontinuous descriptor
GE: Global Encoding
NMBAC: Normalized Moreau-Broto Auto Correlation
QP: Quadratic Programming
LOOCV: Leave One Out Cross validation
ACC: Accuracy
MCC: Matthew’s Correlation Coefficient
SN: Sensitivity
SP: Specificity
AUC: Area under the receiver-operating characteristic curve
ROC: Receiver Operating characteristic Curve
5-CV: 5-fold Cross Validation

## References

[1] Wei L, Tang J, Quan Z (2016). Local-DPP: an improved DNA-binding protein prediction method by exploring local evolutionary information. Inf Sci.

[2] Liu B, Xu J, Lan X, Xu R, Zhou J, Wang X, Chou KC (2014). iDNA-Prot|dis: Identifying DNA-binding proteins by incorporating amino acid distance-pairs and reduced alphabet profile into the general pseudo amino acid composition. PLoS ONE.

[3] Wang Y, Ding Y, Guo F, Wei L, Tang J (2017). Improved detection of DNA-binding proteins via compression technology on PSSM information. PLoS ONE.

[4] Nimrod G, Schushan M, Szilágyi A, Leslie C (2010). iDBPS: a web server for the identification of DNA binding proteins. Bioinformatics.

[5] Bhardwaj N, Langlois RE, Zhao G, Lu H (2005). Kernel-based machine learning protocol for predicting DNA-binding proteins. Nucleic Acids Res.

[6] Cortes C, Vapnik V (1995). Support-vector networks. Mach Learn.

[7] Ahmad S, Sarai A (2004). Moment-based prediction of DNA-binding proteins. J Mol Biol.

[8] Yu X, Cao J, Cai Y, Shi T, Li Y (2006). Predicting rRNA-, RNA-, and DNA-binding proteins from primary structure with support vector machines. J Theor Biol.

[9] Liu B, Xu J, Fan S, Xu R, Zhou J, Wang X (2015). PseDNA-Pro: DNA-binding protein identification by combining Chou’s PseAAC and physicochemical distance transformation. Mol Inf.

[10] Liu B, Wang S, Wang X (2015). DNA binding protein identification by combining pseudo amino acid composition and profile-based protein representation. Sci Rep.

[11] Cai YD, Lin SL (2003). Support vector machines for predicting rRNA-, RNA-, and DNA-binding proteins from amino acid sequence. Biochim Biophys Acta.

[12] Xu R, Zhou J, Wang H, He Y, Wang X, Liu B (2015). Identifying DNA-binding proteins by combining support vector machine and PSSM distance transformation. BMC Syst Biol.

[13] Kumar M, Gromiha MM, Raghava GP (2007). Identification of DNA-binding proteins using support vector machines and evolutionary profiles. BMC Bioinform.

[14] Lipman DJ, Zhang J, Madden T, Altschul SF, Schäffer AA, Miller W, Zhang Z (1997). Gapped BLAST and PSI-BLAST: a new generation of protein database search programs. Nucleic Acids Res.

[15] Lou W, Wang X, Chen F, Chen Y, Jiang B, Zhang H (2014). Sequence based prediction of DNA-binding proteins based on hybrid feature selection using random forest and Gaussian Naïve Bayes. PLoS ONE.

[16] Zou Y, Ding Y, Tang J, Guo F, Peng L (2019). FKRR-MVSF: a fuzzy kernel ridge regression model for identifying DNA-binding proteins by multi-view sequence features via Chou’s five-step rule. Int J Mol Sci..

[17] Ding Y, Chen F, Guo X, Tang J, Wu H (2019). Identification of DNA-binding proteins by multiple kernel support vector machine and sequence information. Curr Proteomics.

[18] Ding YJ, Tang JJ, Guo F (2019). Identification of DNA-binding proteins via fuzzy multiple kernel model and sequence information. Lect Notes Comput Sci.

[19] Liu XJ, Gong XJ, Yu H, Xu JH (2018). A model stacking framework for identifying DNA binding proteins by orchestrating multi-view features and classifiers. Genes.

[20] Rahman MS, Shatabda S, Saha S, Kaykobad M, Rahman MS (2018). DPP-PseAAC: a DNA-binding protein prediction model using Chou’s general PseAAC. J Theor Biol..

[21] Adilina S, Farid D, Shatabda S (2019). Effective DNA binding protein prediction by using key features via Chou’s general PseAAC. J Theor Biol.

[22] Wei L, Luan S, Nagai L, Su R, Zou Q (2019). Exploring sequence-based features for the improved prediction of DNA n4-methylcytosine sites in multiple species. Bioinformatics.

[23] Jia C, Zuo Y, Zou Q (2018). O-GlcNAcPRED-II: an integrated classification algorithm for identifying O-GlcNAcylation sites based on fuzzy undersampling and a K-means PCA oversampling technique. Bioinformatics.

[24] Zeng X, Liu L, Lu L, Zou Q (2018). Prediction of potential disease-associated microrNAS using structural perturbation method. Bioinformatics.

[25] Wei L, Ding Y, Su L, Tang J, Zou Q (2018). Prediction of human protein subcellular localization using deep learning. J Parallel Distrib Comput.

[26] Zou Q, Xing P, Wei L, Liu B (2019). Gene2vec: gene subsequence embedding for prediction of mammalian N6-methyladenosine sites from mRNA. RNA.

[27] Ding YJ, Tang JJ, Guo F (2019). The computational models of drug-target interaction prediction. Protein Pept Lett.

[28] Ding YJ, Tang JJ, Guo F (2019). Identification of drug-side effect association via semi-supervised model and multiple kernel learning. IEEE J Biomed Health Inform.

[29] Ding YJ, Tang JJ, Guo F (2017). Identification of protein-ligand binding sites by sequence information and ensemble classifier. J Chem Inf Model.

[30] Ding YJ, Tang JJ, Guo F (2017). Identification of drug-target interactions via multiple information integration. Inf Sci.

[31] Ding YJ, Tang JJ, Guo F (2019). Identification of drug-target interactions via fuzzy bipartite local model. Neural Comput Appl.

[32] Wang YB, Ding YJ, Tang JJ, Dai Y, Guo F (2019). CrystalM: a multi-view fusion approach for protein crystallization prediction. IEEE/ACM Trans Comput Biol Bioinform.

[33] Jiang L, Xiao Y, Ding Y, Tang J, Guo F (2018). FKL-Spa-LapRLS: an accurate method for identifying human microRNA-disease association. BMC Genomics.

[34] Jiang L, Ding Y, Tang J, Guo F (2018). MDA-SKF: similarity kernel fusion for accurately discovering miRNA-disease association. Front Genet.

[35] Shen C, Ding YJ, Tang JJ, Guo F (2019). Multivariate information fusion with fast kernel learning to kernel ridge regression in predicting LncRNA-protein interactions. Front Genet.

[36] Shen C, Ding YJ, Tang JJ, Jiang LM, Guo F (2019). LPI-KTASLP: prediction of lncRNA-protein interaction by semi-supervised link learning with multivariate information. IEEE Access.

[37] Shen C, Ding YJ, Tang JJ, Xu XY, Guo F (2017). An ameliorated prediction of drug-target interactions based on multi-scale discrete wavelet transform and network features. Int J Mol Sci.

[38] Shen C, Ding YJ, Tang JJ, Song J, Guo F (2017). Identification of DNA-protein binding sites through multi-scale local average blocks on sequence information. Molecules.

[39] Shen YN, Tang JJ, Guo F (2019). Identification of protein subcellular localization via integrating evolutionary and physicochemical information into Chou’s general PseAAC. J Theor Biol.

[40] Ding YJ, Tang JJ, Guo F (2020). Human protein subcellular localization identification via fuzzy model on kernelized neighborhood representation. Appl Soft Comput.

[41] Ding YJ, Tang JJ, Guo F (2020). Identification of drug-target interactions via dual Laplacian regularized least squares with multiple kernel fusion. Knowl Based Syst.

[42] Zhang W, Jing K, Huang F, Chen Y, Li B, Li J, Gong J (2019). SFLLN: a sparse feature learning ensemble method with linear neighborhood regularization for predicting drug–drug interactions. Inf Sci..

[43] Deng Y, Xu X, Qiu Y, Xia J, Zhang W, Liu S (2020). A multimodal deep learning framework for predicting drug–drug interaction events. Bioinformatics.

[44] Ding YJ, Tang JJ, Guo F (2019). Protein crystallization identification via fuzzy model on linear neighborhood representation. IEEE/ACM Trans Comput Biol Bioinform.

[45] Zhang W, Li ZS, Guo WZ, Yang WT, Huang F (2019). A fast linear neighborhood similarity-based network link inference method to predict microRNA-disease associations. IEEE/ACM Trans Comput Biol Bioinform.

[46] Gong YC, Niu YQ, Zhang W, Li XH (2019). A network embedding-based multiple information integration method for the miRNA-disease association prediction. BMC Bioinform.

[47] Zhao Q, Yang YJ, Ren GF, Ge EX, Fan CL (2019). Integrating bipartite network projection and KATZ measure to identify novel circRNA-disease associations. IEEE Trans Nanobiosci.

[48] Liu HS, Ren GF, Chen HY, Liu Q, Yang YJ, Zhao Q (2020). Predicting lncRNA-miRNA interactions based on logistic matrix factorization with neighborhood regularized. Knowl Based Syst.

[49] Zeng X, Lin W, Guo M, Zou Q (2017). A comprehensive overview and evaluation of circular RNA detection tools. PLoS Comput Biol.

[50] Zeng X, Lin W, Guo M, Zou Q (2019). Details in the evaluation of circular RNA detection tools: Reply to Chen and Chuang. PLoS Comput Biol.

[51] Rose PW, Prlić A, Bi C (2015). The RCSB Protein Data Bank: views of structural biology for basic and applied research and education. Nucleic Acids Res.

[52] Lin W, Fang J, Xiao X, Chou K (2011). iDNA-Prot: identification of DNA binding proteins using random forest with grey model. PLoS ONE.

[53] Kumar KK, Pugalenthi G, Suganthan PN (2009). DNA-Prot: identification of DNA binding proteins from protein sequence information using random forest. J Biomol Struct Dyn.

[54] Nanni L, Brahnam S, Lumini A (2012). Wavelet images and Chou’s pseudo amino acid composition for protein classification. Amino Acids.

[55] Cheol Jeong J, Lin X, Chen XW (2011). On position-specific scoring matrix for protein function prediction. IEEE/ACM Trans Comput Biol Bioinform.

[56] Liu B, Liu F, Wang X, Chen J, Fang L, Chou KC (2015). Pse-in-One: a web server for generating various modes of pseudo components of DNA, RNA, and protein sequences. Nucleic Acids Res.

[57] Chou KC, Shen HB (2007). MemType-2L: a web server for predicting membrane proteins and their types by incorporating evolution information through PSE-PSSM. Biochem Biophys Res Commun.

[58] You ZH, Zhu L, Zheng CH, Yu HJ, Deng SP, Ji Z (2014). Prediction of protein–protein interactions from amino acid sequences using a novel multi-scale continuous and discontinuous feature set. BMC Bioinform.

[59] Li X, Liao B, Shu Y, Zeng Q, Luo J (2009). Protein functional class prediction using global encoding of amino acid sequence. J Theor Biol.

[60] Feng ZP, Zhang CT (2000). Prediction of membrane protein types based on the hydrophobic index of amino acids. J Protein Chem.

[61] Ding Y, Tang J, Guo F (2016). Predicting protein–protein interactions via multivariate mutual information of protein sequences. BMC Bioinform.

[62] Chang CC, Lin CJ (2011). LIBSVM: a library for support vector machines. ACM Trans Intell Syst Technol.

[63] Cristianini N, Kandola J, Elisseeff A (2001). On kernel-target alignment. Adv Neural Inf Process Syst.

[64] Cortes C, Mohri M, Rostamizadeh A (2012). Algorithms for learning kernels based on centered alignment. J Mach Learn Res.

